# A solitary osteolytic lesion with pathological fracture in the cervical spine - a case report

**DOI:** 10.1186/s12891-023-06543-2

**Published:** 2023-05-30

**Authors:** Guan-Ming Kuang, Nga-Nuen Loo, Qingpeng Gao, Jishi Li, Lin Luo, Shuang Chen, Jason Pui Yin Cheung, Kenneth M.C. Cheung

**Affiliations:** 1 Department of Orthopaedics and Traumatology, The University of Hong Kong-Shenzhen Hospital, Shenzhen, China; 2 International school, Jinan University, Guangzhou, Guangdong China; 3 Department of Oncology, The University of Hong Kong-Shenzhen Hospital, Shenzhen, China; 4 Department of Radiology, The University of Hong Kong-Shenzhen Hospital, Shenzhen, China; 5 Department of Pathology, The University of Hong Kong-Shenzhen Hospital, Shenzhen, China; 6 Department of Orthopaedics and Traumatology, The University of Hong Kong, Hong Kong SAR, China

**Keywords:** Langerhans cell histiocytosis, Cervical spine, Pathologic fracture, Case report

## Abstract

**Background:**

Langerhans cell histiocytosis (LCH) is a rare disorder. The treatment options vary depending on how many organs are involved and how extensive the disease is. In this report, a case of LCH with isolated 6th cervical vertebra (C6) collapse was presented. This case was treated with anterior corpectomy and instrumented fusion, followed by local radiotherapy (RT), with a good clinical outcome up to postoperative six months.

**Case presentation:**

This was a 47-year-old female patient with a complaint of neck pain and bilateral shoulder pain for two months before consultation. She was initially treated with analgesics, but the pain was persistent. Further radiological evaluations revealed an osteolytic lesion within the C6 vertebral body with a pathological fracture. Magnetic resonance imaging (MRI) with contrast of the cervical spine revealed diffused hypointense signal changes on the T1-weighted images and hyperintense signal changes on the T2-weighted images in the C6 vertebral body, with significant contrast-enhanced infiltration signals. Furthermore, in positron emission tomography-computed tomography (PET-CT), focal hypermetabolism and abnormal uptake signals were seen only in the C6 vertebral body. The patient underwent an anterior cervical corpectomy with instrumented fusion. The histopathological results confirmed the diagnosis of LCH. The patient reported significant pain relief on postoperative day one. Moreover, she was treated by local RT at postoperative one month. Good clinical outcomes were achieved in the form of no pain and recovery in neck mobility up to postoperative six months. No evidence of recurrence was observed at the final follow-up.

**Conclusions:**

This case report describes a treatment option for a solitary C6 collapse with LCH managed by anterior corpectomy and instrumented fusion, followed by local RT, with a good clinical outcome at postoperative six months. More studies are needed to elucidate whether such a treatment strategy is superior to surgery or RT alone.

**Supplementary Information:**

The online version contains supplementary material available at 10.1186/s12891-023-06543-2.

## Background

Langerhans cell histiocytosis (LCH) is a rare neoplasm of hematopoietic myeloid precursor cells that tends to affect Caucasian male children, with a peak incidence around the age of one to three [1]. LCH can present as a single-system or multiple-system involvement. In a report from the International Registry of Histiocyte Society [2], single-system LCH (SS-LCH) in adults was found in 31.4% (81 cases), while 68.6% (188 cases) were found to have multiple systems affected. The most common involvement site in bone is the jaw (29.8%), followed by the skull (21.3%), extremities (17.0%), pelvis (12.8%), vertebrae (12.8%), and ribs (3.4%) [3]. LCH is usually considered a childhood disease. However, Islinger et al. reviewed 541 LCH patients’ records and found that 211 patients (39%) were adults older than 21 years [4]. To diagnose LCH, in addition to clinical findings and radiological investigations, histopathological workups must be performed [5]. In this paper, we describe a case of a 47-year-old female with a solitary LCH lesion in the 6th cervical vertebra (C6) with pathological fracture. The patient was treated with anterior corpectomy and instrumented fusion, followed by local radiotherapy (RT), with good clinical outcomes up to postoperative six months.

## Case presentation

This was a 47-year-old female patient who presented with continuous pain in her neck area and the back of her bilateral shoulders for two months before she came to our hospital for consultation. Her past medical history was healthy, except that a pulmonary nodule had occasionally been seen on a low-dose chest computed tomography (CT) scan. Observation was advised on the nodule, as there were no signs of malignancy. She did not have any family history of malignant tumors or any injury before the onset of neck pain. In addition, she did not have any episodes of fever, significant loss of body weight, or poor appetite after the onset of the pain. The neck pain was mechanical in nature. There was no radiating pain to bilateral upper limbs or numbness over the upper and lower limbs. She was treated in the form of nonsteroidal anti-inflammatory drugs and physical therapy, but the pain persisted and increased in severity over time. On physical examination, a significant reduction in the range of motion of her cervical spine was noted: forward flexion 20 degrees, extension 20 degrees, left and right side bending 25 degrees, and left and right rotation 30 degrees, with marked spasms and tenderness over the paravertebral area. There were no neurological deficits in the upper or lower limbs.

**Fig. 1:**
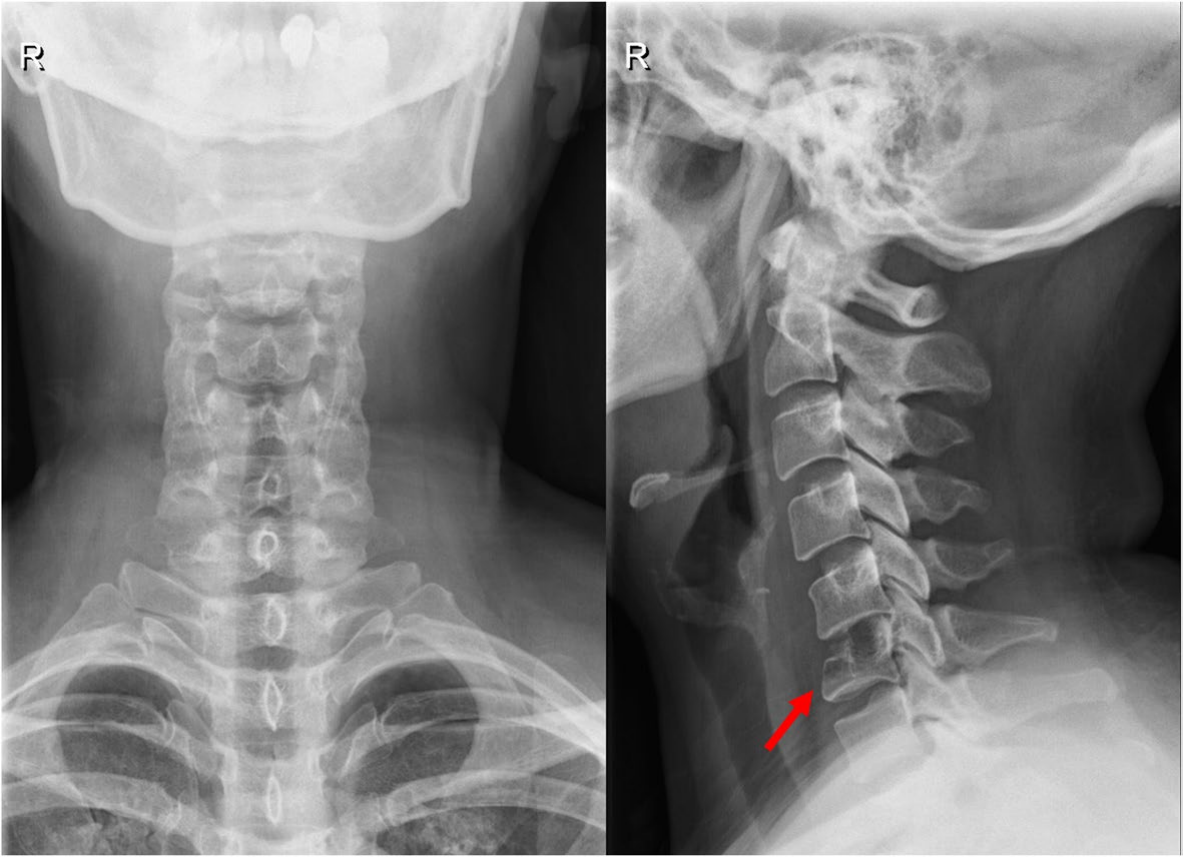
Cervical spine radiograph. **A** anterior–posterior view; **B** lateral view; arrow: C6 vertebral body

**Fig. 2:**
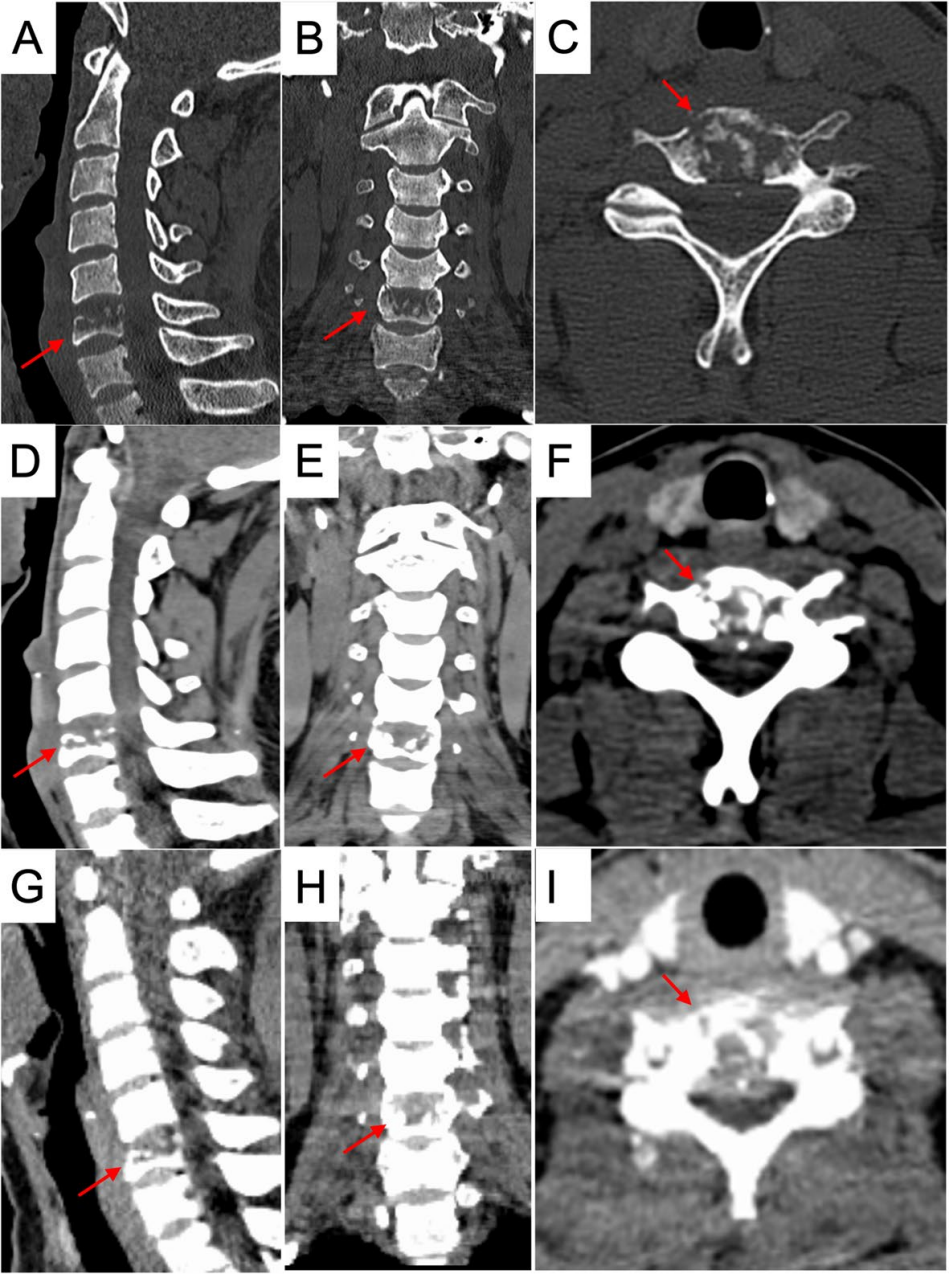
CT scan showing C6 vertebral body collapse and bone destruction. **A** to **C** bone window; **D** to **F** soft tissue window; **G** to **I** contrast-enhancement images; **A**, **D**, and **G** sagittal CT cuts; **B**, **E**, and **H** coronal CT cuts; **C**, **F**, and **I** axial CT cuts; arrows: C6 vertebral body

**Fig. 3:**
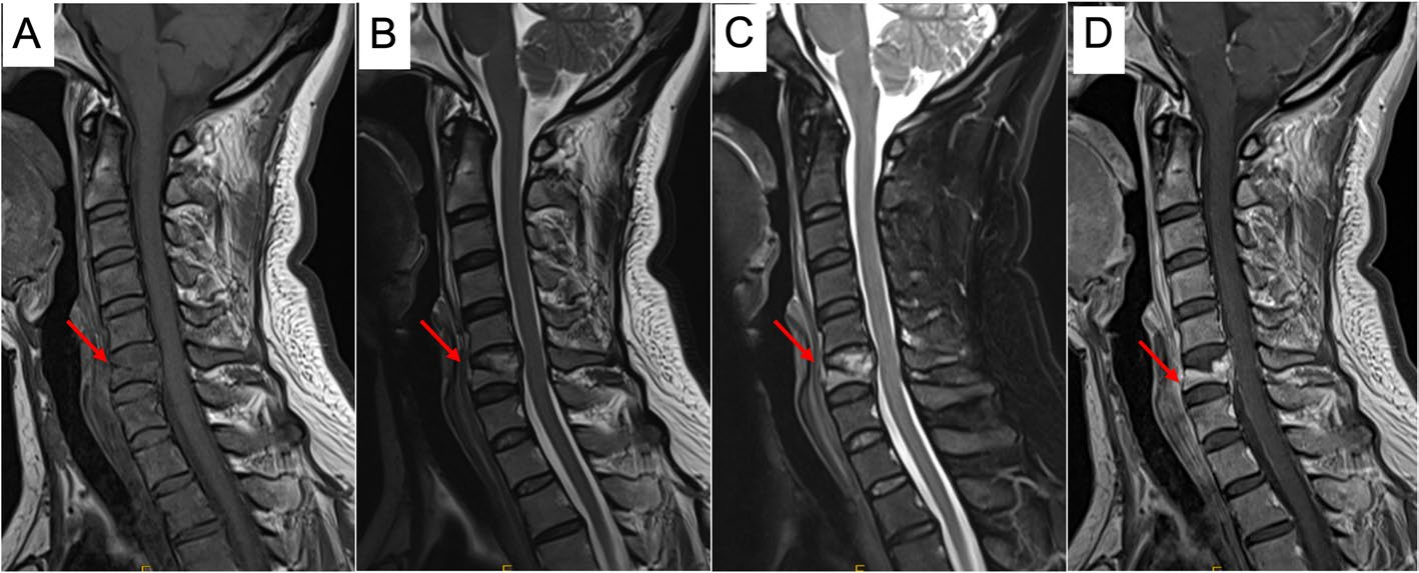
MRI of the cervical spine with contrast (gadolinium), sagittal cut images. **A** T1-weighted image; **B** T2-weighted image; **C** T2-weighted image with fat suppression; **D** Post-contrast T1-weighted image; arrows: C6 vertebral body

**Fig. 4:**
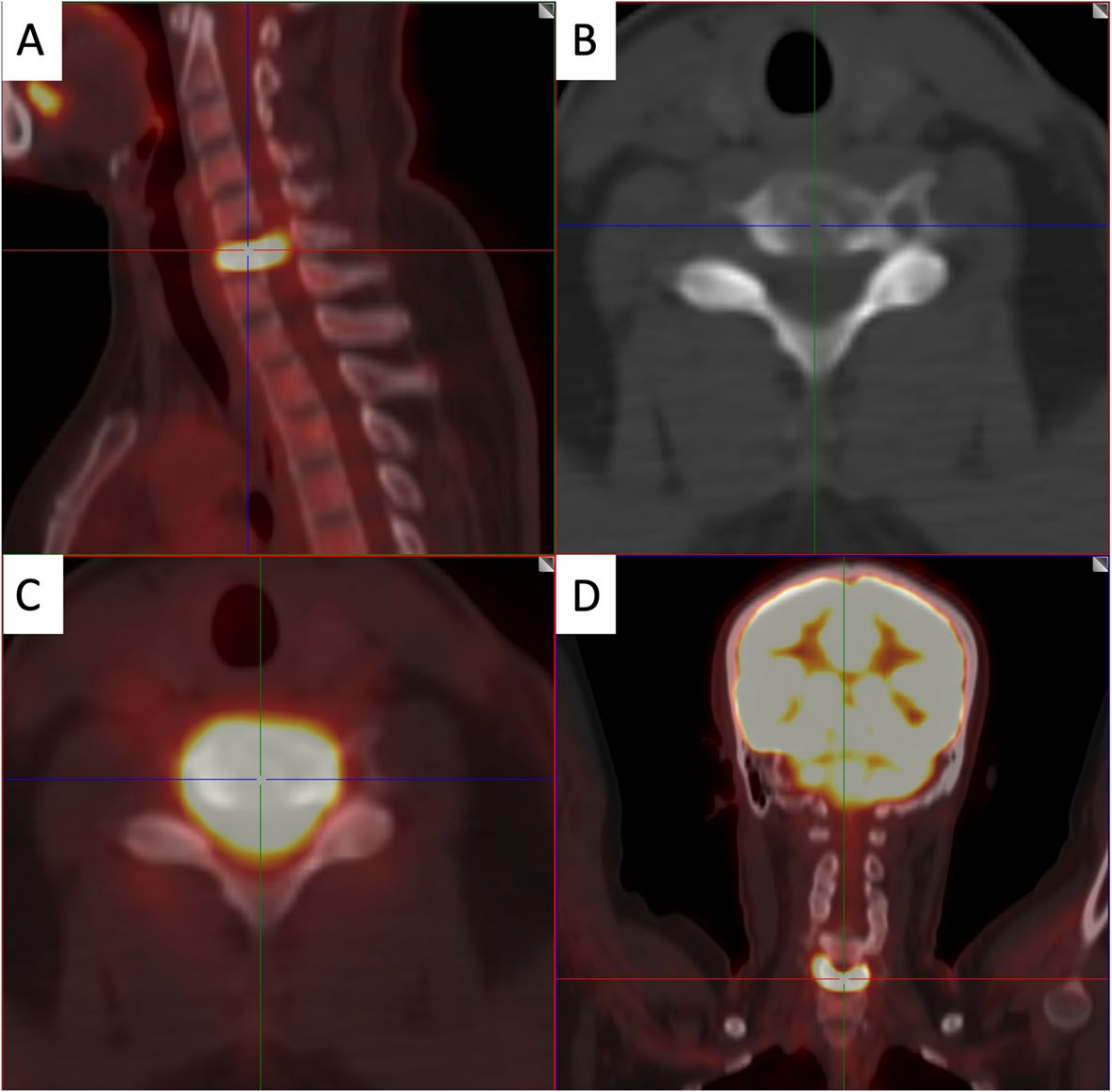
The positron emission tomography-computed tomography (PET-CT). **A** sagittal cut; **B** and **C** axial cuts; **D** coronal cut

For the laboratory data, the complete blood count test, tumor markers, C-reactive protein, and erythrocyte sedimentation rate were all within normal levels. The cervical spine plain radiography revealed that there was collapse in the C6 vertebral body, with mild malalignment in the lateral view. A pathological fracture was considered (Fig. 1). The contrast CT (Fig. 2A to I) showed obvious bone erosion in the C6 vertebral body, involving almost the whole vertebral body, with destructions of the upper endplate and both anterior and posterior walls, leading to a pathological fracture. However, discs at the adjacent levels were not involved. It was also noted that there was no significant enhancement inside the C6 vertebral body (Fig. 2G–I). As both findings of the plain radiography and the CT were unable to provide sufficient information to confirm the patient’s diagnosis, the integrity of the spinal cord, the posterior longitudinal ligament (PLL), the adjacent discs (C5-6 and C6-7), and the paraspinal soft tissues needed to be further assessed; thus, magnetic resonance imaging (MRI) with contrast of the cervical spine was performed. It revealed diffuse hypointense signal changes on the T1-weighted images (Fig. 3A), hyperintense signal changes on the T2-weighted images (Fig. 3B), and T2-weighted images with fat suppression (Fig. 3C) in the C6 vertebral body, with significant contrast-enhanced infiltration signals (Fig. 3D). The signals of the C5-6 and C6-7 discs were relatively normal. Furthermore, in positron emission tomography-computed tomography, focal hypermetabolism and abnormal uptake signals were seen only in the C6 vertebral body, with an SUVmax value of 11.4 (Fig. 4). According to the above clinical and imaging presentations, neoplastic lesions with a pathological fracture of C6 were suspected. Giant cell tumors, LCH, infection, or malignancy, such as primary or metastatic bone tumors, were included in the differential diagnosis. Due to the overlap in the clinical presentations and imaging features of these conditions, the definite diagnoses depended on further investigations, including histopathological and histochemical identification. In addition, tissue cultures of microbes were needed to rule out the possibility of infection.

**Fig. 5:**
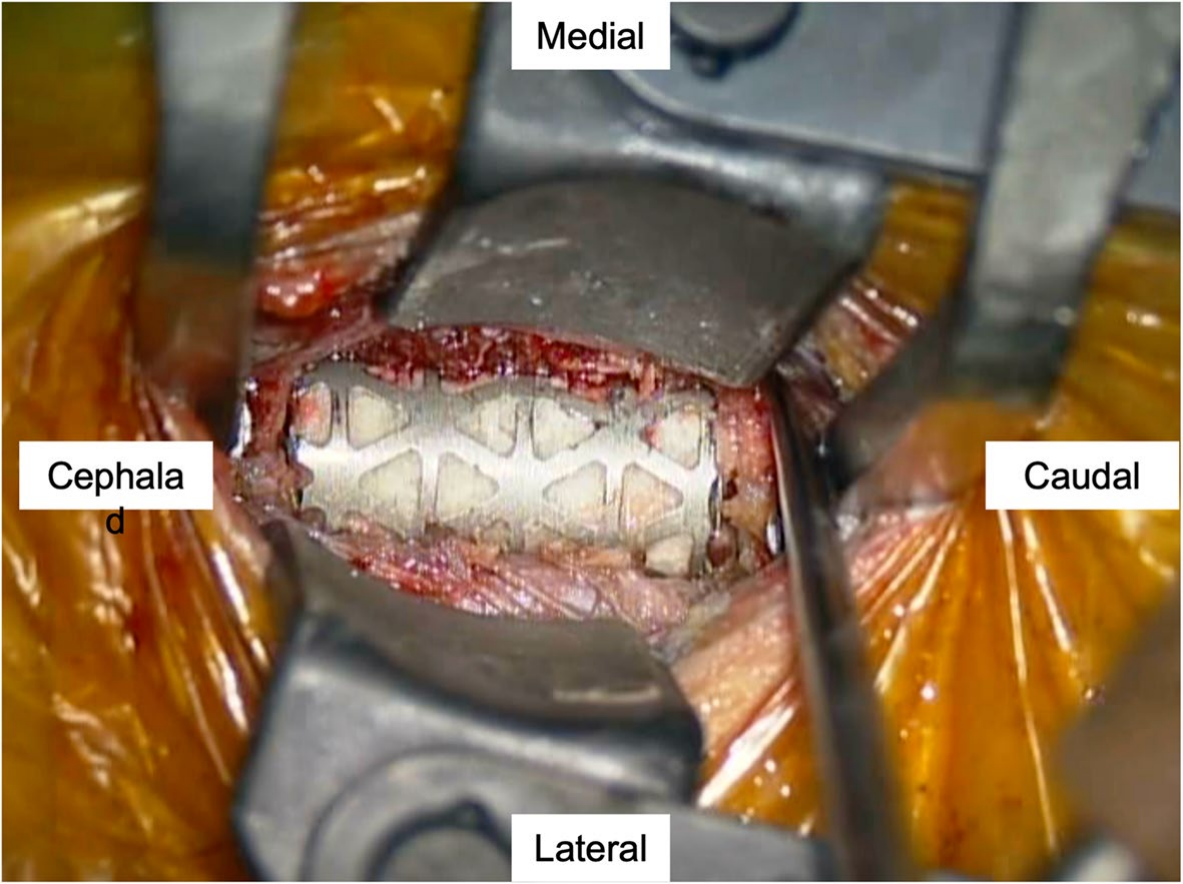
Intraoperative picture showing the titanium mesh cage insertion

**Fig. 6:**
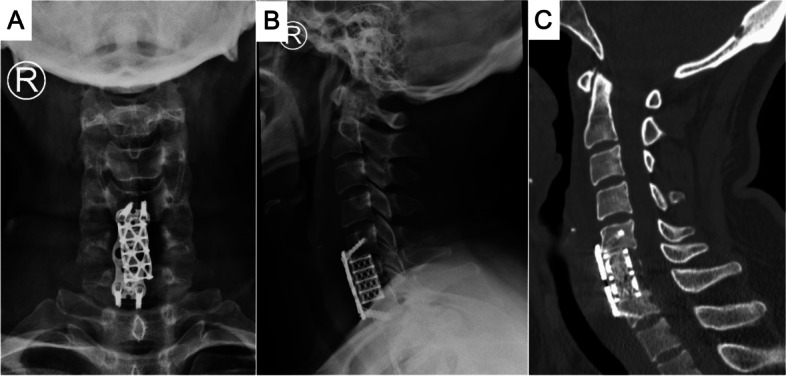
Postoperative images. **A** and **B** cervical spine plain radiography (taken on postoperative day 2); **A** anterior-posterior view; **B** lateral view; **C** CT scan of the cervical spine (taken on postoperative day 4), sagittal cut

**Fig. 7:**
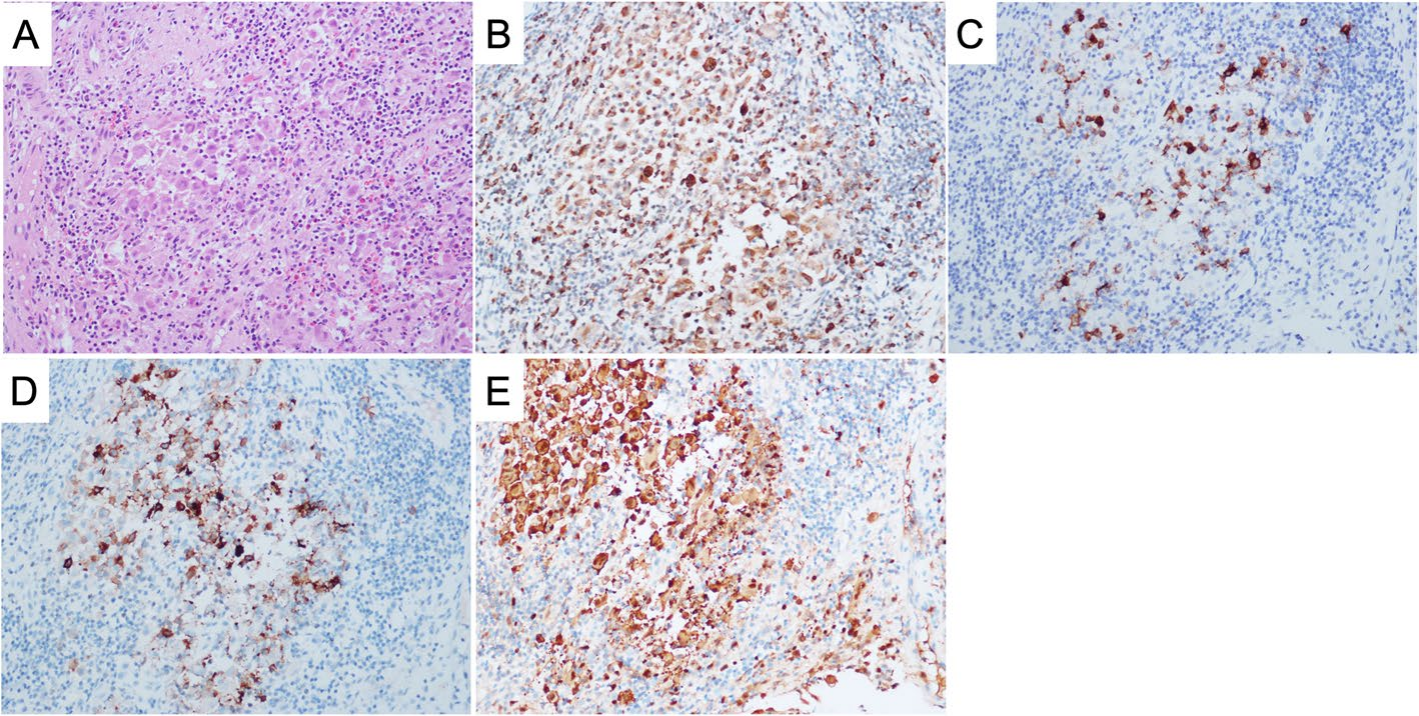
Pathological examination images. **A** hematoxylin–eosin (H & E) staining (original magnification x200); **B** CD68 (original magnification x200); **C**: Langerin (original magnification x200); **D** CD1a (original magnification x200); **E** S-100 (original magnification x200)

Open surgery in the form of a C6 corpectomy with instrumented fusion was performed. The procedure included removing nearly the entire vertebral body (corpectomy) of C6 together with the adjacent discs (discectomy). The resulting bone void after corpectomy and discectomy was filled with bone graft for fusion and stabilized by instrumentation, which included the use of a titanium cage, a plate, and four screws of an appropriate size. Intraoperatively, discectomies of C5-6 and C6-7 were performed. Then, the C6 vertebral body was excised in a subtotal manner. It was observed that the bone lesion involved part of the posterior wall of the vertebral body and part of the PLL. Furthermore, adhesion between the PLL and the dural membrane was noted. The excised tissue was then sent for a frozen section, routine pathology, and tissue culture for microbes. The intraoperative report of the frozen section showed scanty sheets of uniform ovoid cells with eosinophilic cytoplasm. No distinct pleomorphism, mitosis, or necrosis were noted. Such findings favored neoplastic lesions. However, it could not be concluded from the frozen section whether the condition was benign or malignant. Thus, intraoperatively, the decision was made to proceed with an intralesional resection, and the tumor-like tissues were removed as much as possible. A titanium mesh cage filled with granular cancellous bone allograft was inserted between the C5 and C7 vertebral bodies for fusion (Fig. 5). A plate with four screws was applied for fixation. The patient had good recovery in terms of neck and shoulder pain relief on postoperative day one. Postoperative plain radiography showed satisfactory restoration of the cervical spine alignment (Fig. 6A and B). The postoperative CT scan showed that the bone lesions in the C6 vertebral body were excised, and the titanium mesh cage with the filling allograft was in good contact with the lower endplate of C5 and the upper endplate of C7 (Fig. 6C). Gram staining, Acid Fast Bacilli (AFB) smear, aerobic and anaerobic cultures, and fungal cultures were negative. The histology results confirmed the diagnosis of LCH. In the hematoxylin and eosin staining (Fig. 7A) image, tumor cells were characterized by sheets and aggregates in a background of mixed chronic inflammatory cells, particularly eosinophils. The tumor cells were histiocyte-like, with irregular nuclei and abundant cell plasma. Furthermore, there was a positive immunohistochemical reaction of CD68 (Fig. 7B), Langerin (Fig. 7C), CD1a (Fig. 7D), and S-100 (Fig. 7E).

**Fig. 8:**
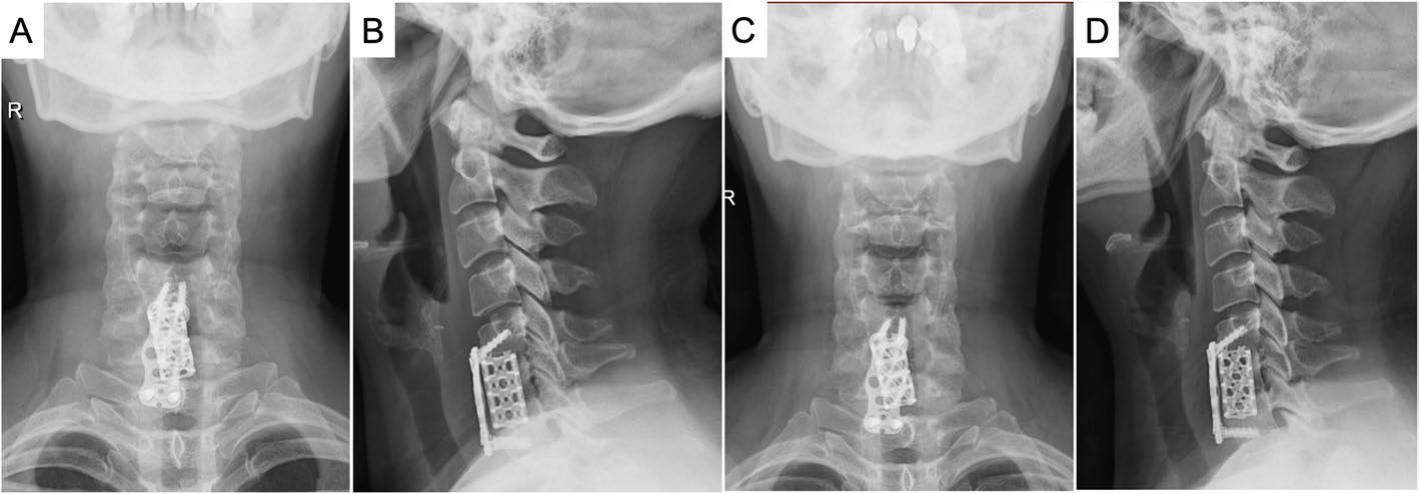
Cervical spine plain radiography taken at postoperative months 2 (**A** and **B**) and 6 (**C** and **D**). **A** and **C** anterior-posterior view; **B** and **D** lateral view

Considering the lesion was close to the cervical spinal cord, and there was adhesion between the PLL and the dural membrane, the patient underwent RT to the C5 to C7 spine (with photon, 6 MV X-ray, volumetric modulated arc therapy, 30 Gy in 15 fractions, 2 Gy per fraction, 5 fractions per week) at postoperative one month, as advised by the oncologist. The patient was followed up at postoperative two months and six months, and showed good clinical outcomes with no pain in the neck and shoulder and a significantly improved range of motion of the cervical spine at last follow-up: forward flexion 40 degrees, extension 35 degrees, left and right side bending 45 degrees, and left and right rotation 60 degrees. Cervical spine plain radiography was repeated (Fig. 8). The cage, plate, and screws were all in position, with acceptable alignment of the cervical spine being restored. There was no evidence of recurrence. The patient had been followed up for half a year, with good recovery in pain at the neck and back of the shoulder.

## Discussion and conclusions

LCH was first described by Paul Langerhans Jr. in 1869 [6]. The first classical case report of LCH was reported by Alfred Hans Jr. in 1893 [7]. LCH refers to a group of pathologies previously known as eosinophilic granuloma of bone, histiocytosis X, Hand-Schuller-Christian syndrome, Letterer-Siwe disease, and self-healing reticulohistiocytosis. LCH can present in several clinical forms, from a lethal leukemia-like disorder that mostly affects infants to a solitary lytic lesion of the bone that is curable [8]. Jean-Francois Emile [5] classified histiocytic disorders into five categories based on their histology, phenotype, molecular alterations, and clinical and imaging characteristics. The five groups of disease are the L group (Langerhans-related), C group (cutaneous and mucocutaneous), R group (Rosal-Dorfman disease), M group (malignant histiocytosis), and H group (hemophagocytic lymphohistiocytosis, and macrophage activation syndrome). LCH belonged to the L group (Langerhans-related), along with indeterminate cell histiocytosis, Erdheim–Chester disease (ECD), and mixed LCH and ECD.

LCH is a rare disease that usually affects children. The age-adjusted incidence rates (0–14 years) were reported to vary between 2.6 [9], 5 [10], and 8.9 [11] new cases per million individuals per year. The incidence of LCH is even more infrequent in adults, with an overall incidence of 0.07 cases per million individuals [12]. LCH is currently classified as an inflammatory myeloid neoplasm driven by activating mutations in the Mitogen-activated protein kinase (MAPK) pathway [1]. The most frequently involved anatomic sites are the bone (38.8%), lung (31.7%), skin (15.4%), lymph nodes (6.3%), mucous membranes (3.9%), and other tissues (3.9%) [2]. The prognosis of this disease depends on the degree of involvement. A poor prognosis is indicated for cases with multiorgan involvement, especially the liver, spleen, or bone marrow [1, 13]. In the spine, as described by Reddy et al. [14], the level of LCH involvement varies in different age groups, with the cervical spine being more frequent in adults (47% cervical, 33% thoracic, and 20% lumbar) and the thoracic spine being more common in the pediatric population (54% thoracic, 35% lumbar, and 11% cervical). In addition, LCH generally involves smaller areas of the vertebral bodies in adults; thus, vertebra plana deformity is less common than in children [14–16]. Furthermore, the two populations also show differences in remodeling capacity, favoring a more conservative approach in young patients [17]. Since the nature of LCH is self-limiting with spontaneous resolution tendency, conservative treatments are encouraged [18]. Surgery is indicated for neurological compromising, spinal instability, and unknown diagnosis [16]. The use of RT in the treatment of vertebral LCH is still controversial and may even be regarded as an overtreatment, as vertebral LCH lesion is considered to be a benign and self-limiting disease.

Pain is a very frequently presented symptom in LCH patients [19, 20]. Huang et al. [21] reported that the most common clinical symptoms of adult LCH patients were pain (83.3%), neurological symptoms (66.7%), restricted motions (58.3%), and deformity (5.6%). In most cases, pain is initially localized and increases in intensity over several weeks. Pain is most prevalent when epidural or bony lesions occur. With the involvement of the nerve root in the cervical or lumbosacral spine, patients usually experience radicular pain, while thoracic spinal lesions are typically accompanied by gripping girdle discomfort. Patients with cervical spinal LCH more commonly reported torticollis or neck stiffness, especially in the upper cervical spine. Although our case showed pain and restricted motions preoperatively, the pain was gone postoperatively. Hence, it is unclear whether the pain was caused by the LCH or the pathological fracture. In this case, the patient did not present with radiating pain to the upper arms or any neurological deficits preoperatively or postoperatively, indicating that there was no neurological involvement before the operation or any complications after the operation.

Plain radiography is important for diagnosing and staging LCH [22]. However, early bone lesions may be neglected by X-rays. In areas at risk of fracture or neurological complications, CT or MRI may enhance the ability to pinpoint the degree of trabecular and cortical bone destruction and guide a bone biopsy if necessary. A study of 41 LCH patients [23] showed different radiological features of LCH in CT scans. In CT scans, diversified types of bone destruction were observed, including geographic (37%), moth-eaten bone destruction with a clear margin (17%), and penetrative (46%) destruction without a clear margin. Of the 41 patients, 29 (71%) had bone cortex destruction, while integrity was compromised. In nine (22%) patients, sclerotic margins were present around the bone destruction. Furthermore, compression fractures were found in 13 patients, including 11 atlas lateral masses and two vertebral bodies in C2. In our patient, the C6 vertebral body was involved. Radiological investigations, including plain radiograph and CT scan, showed osteolytic lesions causing the collapse of the vertebral body. In this case, a core needle biopsy was not performed, as it was not straightforward when trying to reach the anterior portion of the cervical vertebra. In addition, the possible risks associated with anterior cervical spine biopsy include vascular, trachea, esophagus, and recurrent laryngeal nerve injury. Furthermore, the tissue volume received from the needle biopsy may be inadequate, making histopathology inclusive. In this case, with a pathological fracture and instability of the spine, open surgery with internal fixation and fusion was needed. Thus, we considered open surgery in the form of a C6 corpectomy to remove the lesion and instrumented fusion to stabilize the cervical spine.

For adults with LCH, there are no standard therapies, and prospective trials have not been performed [24]. Because LCH can present in heterogeneous ways, treatment options vary depending on how many organs are involved and how extensive the disease is. The treatment of LCH can be categorized into SS-LCH with multifocal or unifocal involvement, or multisystem LCH (MS-LCH) with multiple organ involvement or without risk organ involvement. For unifocal SS-LCH, local therapies are recommended for isolated skin or bone involvement. For MS-LCH and multifocal bone lesion SS-LCH, systemic therapy is strongly recommended. Generally, the treatment modalities are surgical excision, chemotherapy, radiation therapy, or combinations of these treatment modalities [25]. The surgical treatment method usually involves curretage of the bone and lymph node excision. RT can provide local control of LCH involving the bone safely and effectively. Lesions in the bones are often successfully treated with low doses of radiation, whereas diseases in other tissues, such as the skin and brain, may be better treated with higher doses [26].

In a report by Vielgut et al. [16], a similar surgical technique was adopted to treat an adult solitary C5 LCH lesion. A good postoperative outcome in terms of a complete remission of both neck pain and neurologic deficit was achieved immediately post-operation, as well as at the nine-month follow-up. No signs of recurrence were noted at the final follow-up. No postoperative RT was used. However, the bone lesion in the report by Vielgut et al. was relatively limited to the left side of the vertebral body, and no collapse of the vertebral body was presented compared to the case in our current case report [16]. In the report of Dhillon et al. [27], similar procedures, including anterior corpectomy with excisional biopsy and instrumented fusion, were used to treat a solitary 5th lumbar vertebra LCH lesion. The patient reported good early postoperative pain relief. Moreover, a high-dose steroid was given, and there was no recurrence up to two years of follow-up. The case in this report was an SS-LCH case with unifocal involvement in C6. The bone lesion was extensive, leading to vertebral body collapse. The cervical spine was unstable; thus, we performed anterior cervical corpectomy with titanium mesh cage insertion and screw fixation to stabilize the cervical spine. Furthermore, intraoperatively, the adhesion between the PLL and the dural membrane suggests that there was infiltration of LCH to the PLL and the dural membrane. Thus, even though a C6 corpectomy was performed, we could not guarantee that there were no residual LCH tissues. As a result, the patient underwent RT to the C5-C7, intended for better local control of recurrence.

The use of RT in LCH was reported as early as 1979 [28], in which 89 patients with 380 sites of bone lesions received local RT, and over 75% of the sites were controlled. It was also found that nine of 14 patients who received surgical curettage alone had lesions that recurred locally, including one case of vertebral lesion. In contrast, 15 of 16 patients who received radiation therapy showed good local control [28]. In a literature review done by Olschewski et al. [13] that included 683 patients with osseous single-system disease treated by RT alone or combined with other therapies, 658 (96.3%) achieved local control, and 25 (3.7%) experienced local progress. Kotecha et al. [29] retrospectively reviewed 69 patients with RT performed at 169 sites, of which most had bone lesions. Local control achieved 91.4%, and in total, 90.4% of patients had remission of symptoms, with a median follow-up duration of six years. A wide spectrum of doses (2.0–50.4 Gy) was reported for the local control of bone lesions [13, 26, 29], with the most common median doses ranging between 10 and 24 Gy. In this report, the patient was treated with 30 Gy in 15 fractions, 2 Gy per fraction, and 5 fractions per week at postoperative one month. Although the dose was considered high, no radiotherapy-related complications were noted in the patient after therapy. Furthermore, no recurrence was observed at the final follow-up.

In summary, the case of a 47-year-old female with a solitary LCH lesion in C6 with vertebra collapse is reported. Our case differs from the representative cases reported in the literature, in which LCH is generally involved in smaller areas of the vertebral bodies in adults, and vertebral collapse is less common [14–16]. The patient was treated with anterior corpectomy and instrumented fusion due to instability of the cervical spine. Furthermore, postoperative adjuvant radiotherapy was given because of concerns about PLL and dural invasion. We believe that this is the first case in which RT was used as an adjuvant treatment for the spine. The use of radiotherapy in this setting is debatable but represents a possible indication in case of incomplete resection. Moreover, the exact radiation dose has not been established. Although the good clinical outcome up to postoperative six months is shown in this study, a longer follow-up is still needed to observe the long-term results whether there is any local recurrence or any radiation-induced second malignancies. In addition, it is still unknown whether surgery followed by RT leads to lower recurrence rates than either surgery alone or local RT alone for solitary LCH bone lesions. More controlled studies are required with more cases and longer follow-ups to answer these questions. Finally, for vertebral LCH, a core needle biopsy should first be considered as long as it is straightforward to carry out. Injections of steroids are suggested as the first line of treatment when the diagnosis is confirmed. LCH is often a self-limiting disease, and surgery is indicated in cases of neurological symptoms or instability. Radiotherapy is indicated only in selected cases.

## Learning points

- LCH is a rare disorder, and it can present in heterogeneous ways. Treatment options vary depending on how many organs are involved and how extensive the disease is.
- Surgical excision and instrumented stabilization are considered if a solitary lesion leads to bone destruction and instability of the skeleton system (e.g., pathologic fracture in the cervical spine).
- Postoperative radiotherapy is an option intended for better local control of recurrence. However, no evidence has shown the superiority of a strategy in which surgery followed by RT is better than either surgery alone or local RT alone for the treatment of solitary LCH bone lesions.

## Supplementary Information

**Additional file 1.**

## Data Availability

To protect privacy and respect confidentiality, no raw data have been made available in any public repository. The original operation reports, intraoperative photographs, imaging studies, and outpatient clinic records are retained as per the normal procedure within medical records of our institution. The datasets used and/or analyzed during the current study available from the corresponding author on reasonable request.

## Abbreviations

CT: Computed tomography
ECD: Erdheim-Chester disease
ICH: Indeterminate cell histiocytosis
LCH: Langerhans cell histiocytosis
MRI: Magnetic resonance imaging
MS-LCH: Multisystem LCH
PET-CT: Positron emission tomography-computed tomography
PLL: Posterior longitudinal ligament
RT: Radiotherapy
SS-LCH: Single-system LCH
