# Supplementary material for: A solitary osteolytic lesion with pathological fracture in the cervical spine - a case report
Source: BMC Musculoskelet Disord. 2023 May 30;24:436. doi: 10.1186/s12891-023-06543-2 (PMC10228027; doi:10.1186/s12891-023-06543-2)
Supplement: Supplementary file 1 — Additional file 1. [file 12891_2023_6543_MOESM1_ESM.doc]

The Editor

BMC Musculoskeletal Disorders

Dear Editor,

Re: Your submission to BMC Musculoskeletal Disorders -BMSD-D-22-00831R1 - [EMID:e33776e0ece7cbf5]

We would like to re-submit the paper of the title “A solitary osteolytic lesion with pathological fracture in cervical spine - A case report” (BMSD-D-22-00831) for consideration of publication in the Journal. The paper has been amended according to the comments. A clean copy is submitted as requested.

**Editor Comments:**

Thank you for your revisions to date.

May I ask for the following updates in order to move to the next stage.

1- Please re format your Abstract as it begins with the title 'Summary'. Please look at our submission guidelines with regards to sub headings for Case reports.

**Author Response:** Thanks for the comments. The abstract is reformatted according to the guidelines (Page 2 line 2: Abstract). Also, the subheadings are revised accordingly (Page 2 line 3: Background; Page 2 line 8: Case presentation; Page 2 line 21: Conclusions; Page 6 line 1: Discussion and Conclusions).

2- Please submit one clean copy

**Author response:** A clean copy is submitted as requested.
